# Immunological Aspects of Von Hippel-Lindau Disease: A Focus on Neuro-Oncology and Myasthenia Gravis

**DOI:** 10.3390/diagnostics13010144

**Published:** 2023-01-01

**Authors:** Davide Norata, Marta Peri, Giuseppe Roberto Giammalva, Antonino Lupica, Federica Paolini, Lorena Incorvaia, Giuseppe Badalamenti, Valerio Gristina, Antonio Galvano, Antonio Russo, Domenico Gerardo Iacopino, Mauro Silvestrini, Viviana Bazan, Filippo Brighina, Vincenzo Di Stefano

**Affiliations:** 1 Unit of Neurophysiopathology, Department of Biomedicine, Neuroscience and Advanced Diagnostic (BIND), University of Palermo, 90129 Palermo, Italy; 2 Neurological Clinic, Department of Experimental and Clinical Medicine, Marche Polytechnic University, 60121 Ancona, Italy; 3 Unit of Medical Oncology, Department of Surgical, Oncological and Oral Sciences (Di.Chir.On.S.), University of Palermo, 90129 Palermo, Italy; 4 Unit of Neurosurgery, Department of Biomedicine, Neuroscience and Advanced Diagnostic (BIND), University of Palermo, 90129 Palermo, Italy; 5 Section of Medical Oncology, Department of Biomedicine, Neuroscience and Advanced Diagnostics (BIND), University of Palermo, 90127 Palermo, Italy

**Keywords:** VHL disease, immune system, VHL/HIF axis, belzutifan, pazopanib, myasthenia gravis, inflammation, Ach receptors

## Abstract

Von Hippel-Lindau (VHL) disease is an autosomal dominant condition that predisposes affected individuals to a variety of malignant and benign neoplasms. The pathogenetic turning point of this illness is the accumulation of hypoxia-inducible factor (HIF)-1α, a transcription factor of several genes involved in oncogenesis, angiogenesis, tissue regeneration, metabolic regulation, hematopoiesis, and inflammatory responses. From an oncological perspective, increased awareness of the molecular pathways underlying this disease is bringing us closer to the development of specific and targeted therapies. Meanwhile, on the surgical side, improved understanding can help to better identify the patients to be treated and the surgical timing. Overall, pathogenesis research is crucial for developing patient-tailored therapies. One of the actual key topics of interest is the link between the VHL/HIF axis and inflammation. The present study aims to outline the fundamental mechanisms that link VHL disease and immune disorders, as well as to explore the details of the overlap between VHL disease and myasthenia gravis (MG) pathogenetic pathways. As a result, MG becomes a paradigm for autoimmune disorders that might be related with VHL disease.

## 1. VHL Disease: Definition and Pathogenesis

Von Hippel-Lindau (VHL) disease is a hereditary condition with highly penetrant autosomal dominant transmission that predisposes affected people to certain forms of benign and malignant tumors and cysts in a variety of organs (including the kidneys, pancreas, retina, uterus, central nervous system and the adrenal gland). The estimated global prevalence of the disease is between 1 in 31,000 and 1 in 91,000 individuals [1,2,3,4,5,6].

The VHL gene is a tumor suppressor gene sited on the short arm of chromosome 3 (3p25-26) [7]. Affected people usually inherit a germline pathogenic variation of VHL from one affected parent and a normal (wild-type) VHL copy from the other. Consistently with Knudson’s two-hit hypothesis, the first transformed cell of the tumor appears only after the wild-type VHL allele is also inactivated in a cell through a somatic mutation, resulting in the formation of a clonal neoplastic cell that can develop into a tumor mass [8,9].

The VHL gene encodes a 213 amino acid protein (pVHL), which is a part E3 ubiquitin ligase complex and controls the cellular levels of hypoxia-inducible factor (HIF)-1α and HIF-2α, i.e., two recognized oxygen sensors, and thus plays an important role in adaptations to hypoxia [10].

**Figure 1:**
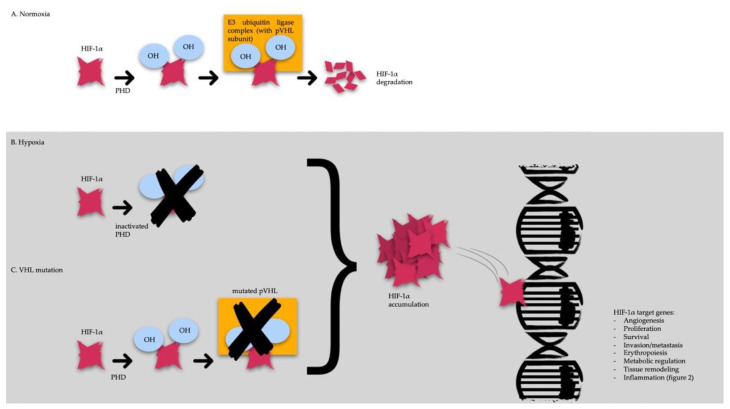
HIF-1α and pVHL function under different conditions: (**A**) in normoxic conditions, HIF-1α is hydroxylated by PHD, bound by the VHL protein complex, and destroyed by the S26 proteasome; (**B**) in hypoxic environments, the inactive PHDs don not hydroxylate HIF-1α, which results in protection from ubiquitination, and hence it tends to accumulate; (**C**) when pVHL is mutated, as in VHL disease, the E3 ubiquitin ligase complex does not work, and HIF subunits accumulate. When HIF-1α levels increase, it modulates the transcription of several genes. pVHL, VHL protein; HIF-1α, hypoxic-inducible factor; PHD, prolyl hydroxylases.

Under normoxic conditions, HIF-1α and HIF-2α are enzymatically hydroxylated by intracellular prolyl hydroxylases (PHD). The hydroxylated HIF subunits are bound by the VHL protein complex, covalently coupled to ubiquitin, and destroyed by the S26 proteasome (Figure 1A) [11,12].

In hypoxia, PHD are inactivated, resulting in a lack of HIF hydroxylation. Non-hydroxylated HIF-1α and HIF-2α are not ubiquitinated by the VHL protein complex and hence tend to accumulate (Figure 1B). Similar events occur when pVHL is mutated, altering the disposal of HIF subunits (Figure 1C). High levels of HIF subunits result in increased transcription of a wide range of genes, including growth and angiogenic factors, intermediate metabolism enzymes, and genes supporting stemness-like cellular phenotypes [13].

## 2. Influence of VHL/HIF Pathway Alterations on Tumor Immune Microenvironment

HIF-1 induces the expression of more than 100 genes, including the vascular endothelial growth factor (VEGF), glycolytic enzymes, and other genes involved in cancer cell survival [14]. Patients affected by VHL syndrome are prone to develop tumors in different organs. Therefore, their treatment implies different subspecialties and a multidisciplinary team [15].

In the era of immune-checkpoint inhibitors in cancer therapy, the key regulator role of VHL/HIF axis of immune cell function increases the oncological research interest and will be discussed in this section.

Hypoxia and the induction of HIF within the tumor microenvironment has been demonstrated to promote cancer progression and treatment resistance by inducing an anti-tumor effector immune suppression and increasing immunosuppressive cells. As demonstrated in other immune-mediated diseases, the microenvironment modifications are characterized by reducing cytotoxic T cells, nature killer cells, and cytokines and increasing Tregs and tumor-associated macrophages owing a M2 macrophage phenotype [16,17].

## 3. Oncological Therapeutical Aspect in VHL Disease

Considering the immunosuppressive impact of HIF in the tumor microenvironment, promoting hallmarks of aggressive cancer behavior such as oncogene activation, drug resistance, and metabolic alterations, it has become as a promising therapeutic target.

Patients with VHL disease have a higher incidence of renal cell carcinoma (RCC), owing to VHL gene inactivation and constitutive activation of the transcription factor hypoxia-inducible factor 2α (HIF-2α). Of interest, our understanding about the role of the VHL/HIF pathway could change the current standard of care in RCC, based on the synergic effect of anti-angiogenic agents and immune checkpoint inhibitors on immunomodulation [18].

The correlation between VHL status and Programmed Death-Ligand 1 (PD-L1) expression has not been extensively investigated, with results being heterogeneous [19].

Recent findings showed that VHL mutant RCC tumors showed a more activated local immunophenotype than wild-type VHL, with increased effector T cells, neoantigen production, and cytokine levels. Whereas in another study, PD-L1 expression was related to wild-type VHL tumors. Molecules that regulate each step of HIF-1α-mediated gene expression in tumor cells have been developed, acting also in other signaling pathways such as the PI3K/AKT/mTOR pathway and VEGFR pathway.

The most promising molecule under investigation is belzutifan (MK-6482, previously called PT2977), an HIF-2α inhibitor that showed activity in patients with RCC and non–RCC neoplasms associated with VHL diseases [20], and a phase 3 trial is planned.

The link between VHL/HIF pathway alterations and the VEGF axis has been demonstrated by encouraging preliminary activity of pazopanib, a multikinase angiogenesis inhibitor, in von Hippel-Lindau disease associated with RCC; retinal, cerebellar, and spinal haemangioblastomas; pheochromocytomas; pancreatic serous cystadenomas; and pancreatic neuroendocrine tumors [21]. The role of pazopanib in this setting is still under investigation.

## 4. Neurosurgery’s Perspective on Patients with VHL Disease

Hemangioblastomas of the CNS are characteristic in VHL patients, often representing the first clinical manifestation of the disease. They are typically multiple, both spinal and cranial. Symptoms and neurological signs are different, from ataxia, dysmetria and slurred speech to paraparesis or quadriparesis, depending on their location [15]. These tumors can affect both children and adults. The pediatric VHL population carries a higher risk of developing obstructive hydrocephalus compared to adults, and cranial pediatric hemangioblastomas can remain clinically occult until the development of severe neurological signs [22,23].

Neurosurgeons’ approaches are different in response to sporadic hemangioblastomas. In the literature, no guidelines about the optimal strategy and surgical timing are available. VHL patients can present multiple lesions from the beginning. When a single lesion is shown, even if total removal of VHL-associated hemangioblastomas is surgically feasible, patients can develop multiple lesions in different areas from the primary region [24].

Previous studies prove that about half of CNS hemangioblastomas remain stable in size, and they do not provoke neurological deficits. This is particularly true when dealing with cranial tumors [22,25].

As regards cranial lesions, there is consensus that only symptomatic tumors should be surgically treated. A watch-and-wait approach is often used in asymptomatic patients, using repeated MRI studies. Treatment strategy can be customized for patients, analyzing tumor location, size, associated cysts, and general conditions of patients. In fact, VHL patients commonly receive several surgeries [22]. As concerns spinal lesions, recent studies show an advantage in early surgery, so defined as surgery in asymptomatic patients, when dealing with lesions around 50 mm^3^ [26,27]. After 5 years, 37% of tumors smaller than 51 mm^3^ and 90% of tumors larger than 51 mm^3^ undergo surgical treatment [26,28].

The aim of surgical treatment is a total removal of the tumor. When not feasible, partial resection is used to preserve surrounding neural structures, together with decompression regarding spinal tumors [22].

Stereotactic radiosurgery (SRS) is an alternative treatment modality for stabilizing tumor growth. It is particularly helpful when dealing with multiple tumors, deep-located tumors, and patients who cannot bear surgical treatment. Prophylactic SRS is not considered a treatment option [22].

## 5. Multidisciplinary Approach for Diagnosis and Surveillance in VHL Disease

Genetic testing, surveillance, and treatment approaches should be handled by a dedicated multidisciplinary team. For diagnosis of VHL disease, red flag manifestations are hemangioblastoma in the retina or the central nervous system, RCC, pheochromocytoma, pancreatic neuroendocrine tumor (PNET), and endolymphatic sac tumor (ELST). Other less common conditions that could support the diagnosis of VHL are pancreatic cysts, kidney cysts, and papillary cystadenoma in the epididymis/papillary cystadenoma of the broad uterine ligament, especially bilateral [29].

In suspected VHL patients, according to latest European guidelines [29], the genetic work-up can be initiated.

Surveillance includes annual focused neurological and retinal examination, hearing examination and annual biochemical screening from age 5 years, and magnetic resonance imaging (MRI) of the abdomen and central nervous system (CNS) every second year from age 15, with a baseline MRI of the CNS at age 10 years [29].

## 6. VHL/HIF Axis in Inflammation and Autoimmunity

The role of HIF-1α in inflammation has been studied in animal models of sepsis, rheumatoid arthritis, and chronic cutaneous inflammation, using conditional gene targeting techniques that allow tissue-specific deletion of HIF-1α or VHL genes. Overall, these investigations found that HIF-1α overexpression causes hyperinflammatory responses and increased vascular permeability, while HIF-1α ablation reduces inflammation [30,31,32,33].

Some investigations on single individuals or small case series have found a possible association between VHL disease and other autoimmune disorders, such as Evans syndrome, which includes autoimmune hemolytic anemia and thrombocytopenia, multiple sclerosis, and autoimmune pancreatitis [34,35,36,37,38,39].

From a cellular and molecular perspective, the VHL/HIF pathway is crucial for the development and function of several immune cells. Lymphoid tissues are indeed constantly exposed to hypoxic challenges under steady and inflamed conditions [40]. Even in the same local environments, such as the germinal center, a gradient of oxygen levels has been observed [41,42,43]. Therefore, the experimental conditions may differ greatly from one study to another (in vitro vs. in vivo, steady state vs. after infection, different metabolic cellular states, etc.), which likely give rise to different biological readouts [40]. Despite these difficulties, a summary of the relevant scientific evidence follows.

### 6.1. The Innate Immune System

The VHL-HIF axis modulates innate immunity since it is required for myeloid cell growth and function [44,45]. Hypoxia can promote the suppressive function of myeloid-derived cells by increasing arginase-1 and inducible nitric oxide synthase (iNOS) production, which is dependent on HIF-1α accumulation [46]. Another study found that low oxygen levels decrease neutrophil apoptosis via HIF-1α-dependent NF-κB (nuclear factor kappa-light-chain enhancer of activated B cells) signaling [47].

In conditional VHL knockout mice, alveolar macrophages (AMs) exhibit immature phenotypes with lower self-renewal capacity due to VHL loss, demonstrating that VHL is essential for AM maturation. Furthermore, VHL deficiency reduces surfactant handling activity by AMs, showing that VHL is responsible for AMs’ ability to remove pulmonary surfactant [48].

Secondly, HIF overexpression in macrophages inhibits localized T cell responses, implying that microenvironmental signals specific to infected tissues may provide a homeostatic mechanism in which myeloid cells and T cells balance effector function to limit excessive tissue damage. This is relevant to cancer and infection; in both cases, the balance of T cell and myeloid cell infiltration in a hypoxic environment can lead to clearance of the diseased tissue or immunosuppression and progression of the pathology [49].

Innate lymphoid cells (ILCs) are a recently characterized subpopulation of lymphocytes that concentrate in peripheral tissues and are particularly prevalent near barrier surfaces. Among them, ILC2s (group 2 ILC) can produce type 2 cytokines in response to alarmin cytokines such as IL-33, IL-25, and TSLP (Thymic Stromal Lymphopoietin), and so play an important role in allergic illnesses, anti-helminth infection, and metabolic balance [50]. A 2018 study on mice that selectively deplete the VHL gene in ILC precursors found that the VHL-HIF-glycolysis axis plays a critical and selective role in the late-stage maturation and function of ILC2s, causing a deficiency of mature ILC2 in peripheral non-lymphoid tissues like the lung, intestine, and lipid tissues and resulting in decreased type 2 immune responses [51].

### 6.2. The Adaptive Cell-Mediated Response

The VHL-HIF pathway is critical for cytotoxic CD8+ T cell development and function. According to a 2013 study, mice with a T cell-specific VHL deletion are more likely than wild-type mice to die from a chronic viral disease (lymphocytic choriomeningitis virus, LCMV), because HIF-1α and HIF-2α overexpression promotes the production of critical transcription factors, effectors, and costimulatory/inhibitory receptors, which improves the function of cytotoxic CD8+ T cells. This represents the failure in the tolerance-exhaustion adaptation to persistent infection [52]. Furthermore, the conditional deletion of VHL accelerates CD8+ memory cell differentiation during viral infections and promotes the formation of long-lived effector-memory CD8+ T cells. This suggests that metabolic modulation by the VHL/HIF axis is essential for the generation of protective CD8+ memory T cells against infections [53].

### 6.3. The Adaptive Humoral Response: B Cells

The hypoxic environment of bone marrow appears to influence hematopoiesis, especially B cell development, as demonstrated in studies that explored how HIF-1α deficiency results in enlarged B1-like cell populations [54,55].

More intriguingly, hypoxia in the germinal center affects B cell antibody production. HIF-1α regulates cell proliferation, survival, and isotype switching [43]. Low oxygen tension or B cell-specific VHL depletion inhibits mTORC1 activity, which is restored by HIF-1α deletion, demonstrating that the VHL/HIF pathway is involved in antibody synthesis in the germinal center during immunological responses [41].

### 6.4. The Adaptive Humoral Response: CD4+ T Cells

Throughout T cell differentiation, hematopoietic progenitor cells travel from bone marrow into the thymus, a main lymphoid organ known to be grossly hypoxic in physiological conditions [51]. High amounts of HIF are actually required for proper thymocyte differentiation [56]. However, when HIF levels rise even higher due to the loss of the VHL gene, caspases are activated, resulting in thymocyte apoptosis [57]. Furthermore, the HIF-1α excess causes an imbalance in calcium transport and, as a result, a calcium-dependent dysfunction of the T cell receptor -TCR (with relative alteration of signal transduction) [58].

The HIF/VHL pathway regulates interferon-γ (IFN-γ) expression in T helper cell type 1 (Th1) immunity during both tumor growth and autoimmune cascades. In VHL-deficient Treg cells, HIF-1α can promote the production of IFN-γ via transcriptional activation by binding to the IFNG gene. In conditional VHL deletion mice, this causes Th1-dependent massive inflammation of multiple tissues [17]. The same process that causes an increase in INF-γ in response to hypoxia appears to produce a greater fragility of Treg cells. Tregs produce a variety of inhibitory cytokines, including transforming growth factor-beta (TGF-β) and IL-10, and are capable of suppressing immunological responses by inhibiting the function of other effector T cells and antigen-presenting cells [59,60]. Their hypoxia-induced fragility is critical for activating the anti-tumor immunity, but at the same time makes the organism more vulnerable to autoimmune processes [61].

In addition, hypoxia is one of a multitude of factors (among them IL-6, TGF-β, and IL-23) [62,63,64] that can influence T lymphocyte development even in peripheral organs. High levels of HIF-1α can indeed imbalance the Th17/regulatory T cell (Treg) ratio, causing a Th17 polarization of the T lymphocyte lineage. In fact, HIF-1α induces the expression of many transcription factors (including the RORγt and STAT3) for Th17, and limits Treg production by downregulating forkhead box protein 3 (FoxP3) [64,65,66].

Th17 cells are crucial in fighting extracellular bacterial and fungal infections in healthy people [67]. These cells, however, can be dysregulated and have harmful roles in autoimmune illnesses such as multiple sclerosis, psoriasis, and rheumatoid arthritis [68,69,70]. Th17 cells’ pro-inflammatory effects are mainly ascribed to the synthesis of cytokines like IL-17, IL-22, IL-17F, and GM-CSF [71,72,73]. These effector molecules and other substances contribute to inflammation by neutrophil recruitment, B cell function enhancement, activation of innate immune cells, and induction a cascade of more pro-inflammatory cytokines [74,75].

In Th17 cells, pVHL also regulates several biological pathways, including genes encoding proteins involved in glycolysis, which, from a metabolic approach, is critical to the function of these cells. Therefore, even if represented in greater number, VHL-deficient Th17 cells present a differentiation defect that cannot be attributed to increased cell death, a tendency to develop into Treg cells, or a lack of IL-23 signaling. These cells show instead a harmful increase in glycolysis, without any alteration in oxidative phosphorylation. This observation has significance for the development of therapies for autoimmune disorders targeting the immune-metabolic system [76,77].

## 7. VHL Disease and Myasthenia Gravis

Myasthenia gravis (MG) is an acquired autoimmune neurological condition that causes defective transmission in the postsynaptic membrane at the neuromuscular junction [78,79]. Antibodies against acetylcholine receptors (AChR), muscle-specific kinase (MuSK), and lipoprotein receptor-related protein 4 (LRP4) are among the pathogenic antibodies that can be currently detected in myasthenic patients [80]. Nowadays, several more antigenic targets have been identified, and the corresponding antibodies can be detected either alone in “seronegative” individuals or in combination with the aforementioned antibodies. These targets include titin and other muscle fiber proteins (ryanodine receptor, actin, myosin, tropomyosin, filamin, etc.), agrin, Kv1.4 potassium channel, rapsyn, cortactin, acetylcholinesterase, collagen Q, and collagen XIII [81].

Table 1 displays two previously published clinical examples of VHL disease in association with Myasthenia Gravis, as well as two additional cases recently observed in our clinic [82,83]. It would be fascinating to investigate if these individuals have a particular antibody profile other than the anti-AChR ones, and to study their potential pathogenicity in this specific setting.

These cases may indicate random connections between VHL disease and MG, but they require scientific consideration due to the complex function that pVHL deficiency has in the pathogenesis of autoimmune illnesses.

### 7.1. Myasthenia Gravis Autoimmune Pathways

In MG patients, IFN-γ and IL-17 blood levels are elevated, suggesting a role for Th1 and Th17 cells in MG pathogenesis. Furthermore, Treg’s ability to block T cell responses is severely hampered [84].

The underlying mechanisms of these alterations are still not completely known, but anti-AChR MG is often associated with thymic dysfunction, with thymic hyperplasia being the most frequent [85]. The development of germinal centers in the thymus is a common pathogenic alteration in MG patients [86]. In the case of early-onset anti-AChR MG, the thymic germinal centers overexpress pro-inflammatory cytokines and thymic epithelial cells in response to AChR stimulation, presenting AChR subunits to autoreactive CD4+ T cells (Th1), thereby upregulating IL-4 and IL-6 and stimulating B cell proliferation to promote the production of anti-AChR antibodies [87]. Indeed, B cell–depleting therapies are significantly effective in AChR-positive MG.

Several investigations have shown that Th17 cells and IL-17 are linked to MG severity. In two studies on IL-17 knock-out mice, the authors detected reduced myasthenia symptoms and large decreases in anti-AChR antibodies, suggesting that IL-17 regulated the B-cell antibody generation, and supporting the involvement of Th17 cells in MG autoimmunity [88,89].

Th17 cells have a major impact on the Th1/Th2 cytokine balance, influencing antibody production in MG patients [90]. As a result, Th17 cells and the cytokines they release have been related to the generation of anti-AChR antibody-mediated autoimmunity at the neuromuscular junction. Therefore, it is not surprising that serum IL-17 levels have been associated with the degree of quantitative MG scores and anti-AChR antibody titers, indicating a more severe illness course [91].

Most investigations have revealed Tregs reduction in number or the presence of functional abnormalities in MG patients. The number of CD4^+^CD25^+^FoxP3^+^Tregs in peripheral blood decreases during the more severe stages of anti-AChR antibody-positive MG and increases with treatment [92], with significantly lower levels in generalized anti-AChR MG without thymoma than in ocular MG [93].

Furthermore, other investigations have shown a significant functional impairment in Treg regulatory activity in MG individuals, with alterations in the inhibitory function of responder T (Tresp) cells mediated by Tregs and a drop in FoxP3 expression, as previously documented [92,94,95,96]. As a result, functional impairment of Tregs in the periphery and thymus is more likely to be caused by MG.

However, two studies on T regs seem to contradict these findings. The first of these studies shows no discernible differences in the relative proportion of Tregs between MG patients and healthy controls [94]. On the other side, Huang et al. revealed that when activated with IFN-γ, CD4^+^CD25^-^T-cells from MG patients changed into CD4^+^CD25^+^Tregs expressing FoxP3 [97]. Notably, these two investigations identified Tregs based on high CD25 expression, although CD25 was also expressed in other T cells (including effector T cells or T cells with pro-inflammatory features) [98].

### 7.2. Possible Interactions between Myasthenia Gravis and Von Hippel-Lindau Disease Immunological Pathways

**Figure 2:**
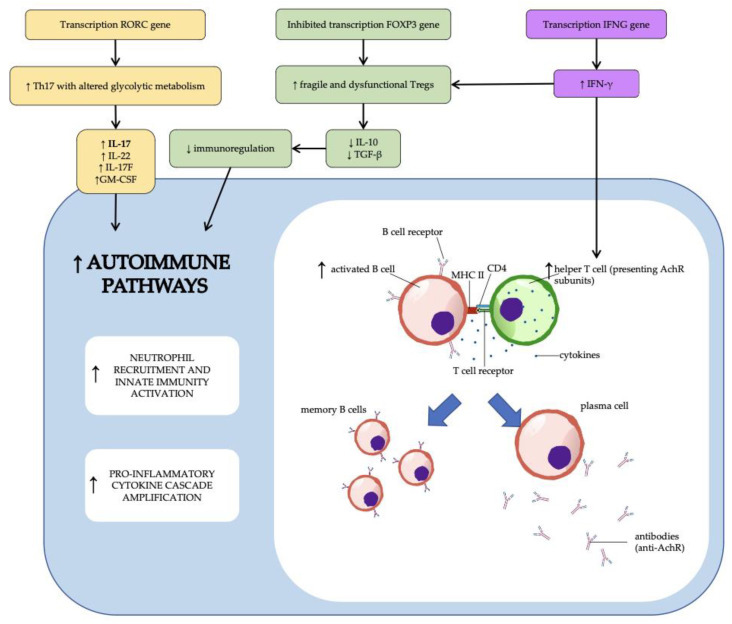
Immune system-related genes activated/inhibited by HIF-1α transcriptional function in hematopoietic progenitors. RORC (RAR-related orphan receptor C) gene encodes for RORγt, FOXP3 (forkhead box protein 3) gene encodes for FoxP3, and IFNG gene encodes for INF-γ.

According to the available evidence, we can postulate that there might be a pathogenetic overlap between VHL disease-related autoimmunity and Myasthenia Gravis. This relationship seems to mainly involve the adaptive immunity system, in both its humoral and cell-mediated components, and operates on three levels, corresponding to three cell lines that are important in the pathophysiology of both conditions: Th1, Treg, and Th17 lymphocytes (Figure 2).

In patients with VHL disease, increasing HIF-1α levels cause the activation of the *IFNG gene*, resulting in accumulation of IFN-γ [17]. This cytokine is critical in determining the numerical expansion of Th1 cells, which more easily present AchR subunits to B cells, resulting in an increase in antibody production (in particular anti-AchR antibodies) [87].

Rising levels of IFN-γ in VHL-deleted people are also linked to increased fragility of Tregs, which are dysfunctional and no longer contribute to immune system modulation [61]. This mechanism is necessary for the immune system to react against the neoplastic cells of VHL disease, but it is also harmful since it contributes to autoimmunity. Myasthenic patients with these Treg alterations present more severe symptoms and a generalized type of the disease [92,93].

The boost of HIF-1α in blood progenitors of patients with VHL mutation also shifts the lymphocytic patrimony toward Th17 cells, with an altered glycolytic metabolism [64,65,66]. These cells synthetize, among other cytokines, IL-17, whose blood levels are especially high in myasthenic patients. IL-17 and other Th17-interleukins support neutrophil recruitment, innate immunity activation, cytokine cascade amplification, and B cell activity [74,75]. This phenomenon may be responsible for the directly proportional link between IL-17 levels, antibody titer rising, and severity of symptoms in myasthenic patients [91]. Furthermore, IL-17 shifts the Th1/Th2 cells ratio in favor of the numerator, implementing the antigen presentation pathway outlined above [90].

## 8. Conclusions

Von Hippel-Lindau disease, while being caused by a specific gene mutation, is a complex illness involving several systems and structures. The VHL/HIF axis does, in fact, impact the transcription of numerous genes implicated in various mechanisms, including oncogenesis and autoimmunity. In the age of targeted and personalized medicine, with anti-angiogenic agents, immune checkpoint inhibitors, and gene silencers (among other treatments), a thorough comprehension of what is going on at the molecular level is critical. The ultimate goal is to identify variables that can result in a patient-tailored treatment.

The present study summarizes the main mechanisms that link VHL disease and autoimmunity, as well as provides an overview of the overlap between the pathogenetic pathways of VHL disease and myasthenia gravis, using MG as a model of autoimmune disorders not yet associated with this hereditary illness. More specifically, in MG pathophysiology, hypoxia due to respiratory muscle exhaustion might cause further HIF-α accumulation, amplifying the processes.

The phenomena presented help to better explain why there are patients with both VHL disease and MG. More broadly, they may aid in understanding how the VHL/HIF axis influences the autoimmune cascade. To fully comprehend the relevance of this potential link, further research is required to actively look for evidence of autoimmune disorder, even if subclinical, in individuals with VHL disease.

## Figures and Tables

**Table 1:** Clinical features of patients with VHL disease and MG.

Case Report	Sex, Age (y) VHL Mutation	VHL-Related Tumors	MG Symptoms/Signs	**1.** **Suggestive 3 Hz RNS Findings** **2.** **Elevated Anti-AchR Ab**	Thymic Disease (Histological Diagnosis)
Sheth et al., 2005 [82]	M, 47 -	Cerebellar hemangioblastoma Retinal angioma Renal cysts RCC	Right ptosis, binocular diplopia Dysarthria, dysphagia, exertional dyspnea, mild facial weakness Proximal lower extremity bilateral weakness	YES YES	Thymoma
Pozzato et al., 2009 [83]	F, 60 New VHL mutation (c279delC) + polymorphism c291C > G	Cerebellar hemangioblastoma Pancreatic cyst RCC	Left ptosis, binocular diplopia Dysarthria Bilateral upper and lower limbs weakness	YES NO	-
Present case 1	F, 17 Paternal VHL mutation	Spinal hemangioblastoma	Left ptosis, binocular diplopia Sporadic dysarthria	– YES	Thymic hyperplasia
Present case 2	M, 51	Cerebellar hemangioblastoma	Binocular diplopia Sporadic dysphagia Lower limbs weakness	NO, (but suggestive SFEMG) YES	-

RCC, clear cell renal carcinoma; VHL, von Hippel Lindau; MG, myasthenia gravis; anti-AchR Ab, antibodies against acetylcholine receptor; SFEMG, single-fiber electromyography.

## Data Availability

Data are available from the corresponding author upon a reasonable request.
